# Risk factors for carriage of *Streptococcus pneumoniae* in children

**DOI:** 10.1186/s12887-018-1119-6

**Published:** 2018-04-26

**Authors:** Maria G. Koliou, Kyriaki Andreou, Demetris Lamnisos, Giagkos Lavranos, Paris Iakovides, Christos Economou, Elpidoforos S. Soteriades

**Affiliations:** 1 Department of Paediatrics, Archbishop Makarios III Hospital, Nicosia, Cyprus; 2 School of Medicine, University of Cyprus, Nicosia, Cyprus; 3 Cyprus Institute of Biomedical Sciences (CIBS), Nicosia, Cyprus; 4 The Hull IVF Unit, Hull, UK; 5 Department of Health Sciences, School of Sciences, European University Cyprus, Nicosia, Cyprus; 6 Private practice, Nicosia, Cyprus; 7 Harvard School of Public Health, Department of Environmental Health, Environmental and Occupational Medicine and Epidemiology (EOME), Boston, USA

**Keywords:** *Streptococcus pneumoniae*, Colonization, Risk factors, Drug resistance, Vaccination, Cyprus

## Abstract

**Background:**

During the past decades *Streptococcus pneumoniae* has developed significant resistance to many classes of antimicrobial drugs. Potential risk factors for colonization of the nasopharynx by *Streptococcus pneumoniae* in children and for carriage of drug resistant strains were examined.

**Methods:**

Between 2007 and 2008 nasopharyngeal swabs were collected from 402 children 6 months to 5 years old visiting the public sector immunization centers and outpatient departments as well as offices of paediatricians from private practice in Nicosia district in Cyprus. Information on demographic characteristics and potential risk factors of participating children were collected using a standardized questionnaire distributed to parents.

**Results:**

In multivariable analyses we found that attendance at day care center, having siblings in the family and having both parents originating from Cyprus, statistically increased the risk of pneumococcal colonization. Full immunization with PCV7 appears to be a protective factor against colonization by pneumococcus. Previous administration of antimicrobials during the last month prior to specimen collection appeared to be the most consistent risk factor for carrying a non susceptible strain of *Streptococcus pneumoniae* to either penicillin or erythromycin. Factors such as age, nationality, previous or current breastfeeding, passive exposure to cigarette smoke and attendance in a day care center do not appear as independent risk factors for colonization by non susceptible strains.

**Conclusions:**

Prudent use of antibiotics especially for upper respiratory tract infections in children as well as increased vaccination coverage by the pneumococcal conjugate vaccines could prove effective in reducing levels of colonization by drug resistant pneumococcal strains.

## Background

*Streptococcus pneumoniae* is a common cause of meningitis, pneumonia and bacteraemia particularly among children and the elderly. It is also the most frequent pathogen causing acute otitis media in children and the most frequent bacterial cause of disease in children younger than 3 years of age [1]. Colonization of the nasopharynx by *S. pneumoniae* is considered a prerequisite for invasive disease and has a key role in the transmission of the pathogen to other individuals leading to pathogen spread in the community [2, 3]. Therefore strains colonizing the nasopharynx reflect the strains circulating in the community leading to different infections [4].

During the past decades, global development of resistance of *S. pneumoniae* to several antimicrobials appears to pose a significant challenge to the successful treatment of infections [5]. Development of pneumococcal conjugate vaccines has led to a significant decrease of pneumococcal infections in general and also to infections caused by drug resistant strains [6]. Conjugate vaccines act by decreasing infections caused by vaccine serotypes. They also reduce colonization by the same serotypes; a result which appears to account for a significant herd effect noted in older age groups who have not been vaccinated [7].

Vaccine serotypes account for the highest percentage of resistance in most countries. Several factors appear to influence the type and frequency of pneumococcal isolates colonizing the nasopharynx. Environmental and socioeconomic factors, overcrowding conditions and overconsumption of antimicrobials have been reported in various studies [8–11].

In Cyprus, the PCV7 (Pneumococcus 7-valent conjugate vaccine) was introduced in 2004 only in the private sector and was not subsidized by the state until 2010 when the 10-valent vaccine was introduced in the public sector and was offered free of charge. In 2011 the 13-valent vaccine was also introduced in Cyprus but only by the private sector. Therefore population coverage of children by the PCV7 vaccine in 2007–2008 when the current study was conducted remained relatively low. Data from the tri-annual survey performed in Cyprus by the Ministry of Health among children 17–24 months old in 2009, revealed full coverage by the PCV7 vaccine only in 37.5%, partial coverage in 42% and no vaccine coverage in 20.5% of children [12]. The susceptibility pattern and the circulating serotypes of *S. pneumoniae* isolated in Cypriot children have been reported elsewhere [13]. So far, no previous studies have been reported in Cyprus examining potential risk factors for colonization by pneumococcus in Cypriot children. The objective of our study was to evaluate the potential risk factors among Cypriot children associated with colonization of nasopharynx by *S. pneumoniae* and those risk factors that may be associated with the carriage of drug resistant strains.

## Methods

### Design and setting of the study

The study was conducted in Nicosia district (capital); the largest district of Cyprus, between November 2007 and May 2008. The population of Nicosia district at the end of 2007 was 301,103 people representing 38.8% of the total population of the Republic of Cyprus. The target population of the study was children 6 months to 5 years old, who visited the government immunization centers in urban and rural areas, the outpatient department of Archbishop Makarios III Hospital or those who visited the offices of 4 major pediatric units of the private sector. Due to the absence of a National health care system in Cyprus, health services are currently offered independently in the public and private sector. The public sector in Cyprus provides health coverage to about 80% of the population, which is eligible for medical care, including current and former public sector employees and their families, individuals with low income and other EU (European Union) nationals residing in Cyprus with recognized medical coverage in their country of origin. On the other hand, the private health sector in Cyprus currently offers its services to a particular segment of the population, who either pay out of pocket or maintain coverage from private health insurance plans. For each child participating in the study, written informed consent was taken from the parents or legal guardians. The study was approved by the National Bioethics Committee of the Republic of Cyprus.

Epidemiological and demographic data were collected through standardized questionnaires which were addressed to the parents at the time of specimen collection. Information was gathered on age, gender, parent nationality, exposure to passive smoking, previous or current breastfeeding, number of siblings, attendance to day care centers, immunization with PCV7 vaccine, current illness, whether the sample was taken from a public sector center or from a private paediatrician’s office (specimen origin) and on recent use of antibiotics. Children suffering from a bacterial infection or being on antibiotics at the day of specimen collection were excluded from the study. Specimens were taken from only one child from each family.

### Sampling procedure

Nasopharyngeal specimens were collected using sterile Rayon swabs on flexible wire (Medical Wire and Equipment Co. Ltd.,Corsham, Wilts., England) and were plated within two hours on Blood agar plates supplemented with 5% defibrinated horse blood and chocolate agar plates and incubated in 5% CO_2_ for 24 h at 35 °C. *S. pneumoniae* strains were identified by optochin sensitivity and bile solubility as described before [14].

### Susceptibility testing

Susceptibility testing was performed on Mueller-Hinton agar supplemented with 5% defibrinated horse blood as follows: All strains were tested by the disk diffusion methods for susceptibility to oxacillin, cefotaxime, erythromycin, clindamycin, trimethoprim – sulfamethoxazole, tetracycline, vancomycin, linezolid and levofloxacin and by assessing the MIC (Minimum Inhibitory Concentration) of all strains to penicillin. MIC was determined by the Etest method (AB Biodisk, Solna, Sweden) [15]. MIC was also determined for all antibiotics to which *S. pneumoniae* appeared non susceptible by the disk method.

Subsequently, the results of susceptibility to penicillin were interpreted based on the new definitions issued by the CLSI (Clinical and Laboratory Standards Institute) at the beginning of 2008 as applied for a non-meningitis infection if antibacterial is administered orally [16]. A strain is considered sensitive to penicillin if MIC is < 0.06 μg/ml, intermediate resistant if MIC is between 0.12–1 and resistant if MIC is > = 2 μg/ml.

### Statistical analysis

The following factors were examined as possible risk factors for colonization and carriage of drug resistant strains for *S. pneumoniae* in the children population: gender, age, parent nationality, exposure to passive smoking, previous or current breastfeeding, number of siblings, attendance to day care centers, immunization with PCV7 vaccine, whether the sample was taken from a public sector center or from a private paediatrician’s office (specimen origin) and on recent use of antibiotics. These factors were first analyzed by a series of univariable analyses. Then, to control simultaneously for the possible confounding effects of the different variables, the risk of colonization and carriage of drug resistant strains of *S. pneumoniae* was estimated by multiple logistic regression analyses that simultaneously included all factors as predictor variables in the model. In both analyses, the association was expressed in OR (odds ratios) and 95% CI (confidence intervals). Statistical analyses were performed using the statistical package SPSS 20. Statistical significance was set at *p* < 0.05.

## Results

### Demographics

One nasopharyngeal specimen was taken from each of 402 children who represented 2.4% of the total population of the children of same age in the district of Nicosia. In Table 1 we present the socioeconomic and demographic characteristics of the children enrolled in the study. A total of 143 children attended a variety of 19 different day care centers while 33 children were cared for during the morning hours by an adult who also provided care for a small number of other children. The majority of children (226) stayed at their home supervised by an adult family member. A total of 60.3% of the specimens were obtained from children visiting a public sector center and 39.8% from the offices of private paediatricians. We found that 35.3% of the children in the sample were colonized by *S. pneumoniae.*

**Table 1:** Sociodemographic characteristics

Characteristics/variables	Categories	Frequency (%)
Gender	Boy	207 (51.5)
	Girl	195 (48.5)
Age	< 1 year	130 (32.3)
	1–2	123 (30.6)
	> 2 years	149 (37.1)
Nationality	Both parents Cypriots	273 (67.9)
	Others	128 (31.9)
	Missing	1(0.2)
Breastfeeding	Yes	300 (74.6)
	No	97 (24.1)
	Missing	5 (1.2)
Exposure to smoking	Yes	142 (35.3)
	No	255 (63.4)
	Missing	5 (1.2)
Day care attendance^a^	Yes	170 (42.3)
	No	226 (56.2)
	Missing	6 (1.5)
Siblings	No	168 (42.5)
	1	140 (35.4)
	> = 2	87 (22.0)
Vaccinated	Full	91 (22.6)
	Incomplete	53 (13.2)
	No	226 (56.2)
	Missing	32 (16)
Origin of specimen	From a public center	241 (60.3)
	From the private sector	159 (39.8)
Colonized	Yes	142 (35.3)
	No	260 (64.7)

^a^Includes children who were supervised during the morning hours by an adult who also cared for at least 3 other children

### Risk factors for carriage

In Table 2 we delineate the association of different risk factors with the colonization of nasopharynx of children by *S. pneumoniae*. In multivariable analyses, we did not find any association between age or gender and the risk of colonization with *S. pneumoniae*. Furthermore, we did not find any statistically significant association between history of breastfeeding, exposure to passive smoking and specimen origin (obtained from a child attending the public sector versus the private sector) and the risk of colonization. Day care attendance or having siblings (one or more) appeared to increase the risk by nearly 3 fold. If both parents were Cypriot nationals in comparison with any other nationality also appeared to increase the risk of colonization by more than two times. On the other hand, full immunization status by PCV7 was associated with a protective effect (two-fold lower risk).

**Table 2:** Association between potential risk factors and colonization by pneumococcal isolate

Variable/risk factor	Category	Percentage of colonized children (%)	Unadjusted OR (95% CI)	Adjusted OR^d^ (95% CI)	*p*-value^¶^
Gender	Boy	37.7%	Ref ^a^	Ref ^a^	
	Girl	32.8%	0.81 (0.54, 1.22)	0.91 (0.57, 1.47)	0.71
Age	< 1 year	26.2%	Ref	Ref	
	1–2	30.9%	1.26 (073, 2.19)	0.93 (0.50, 1.74)	0.83
	> 2 years	47.0%	**2.50 (1.52, 4.18)** ^c^	1.31 (0.63, 2.72)	0.47
Nationality	Both parents Cypriots	41.0%	**2.27 (1.43, 3.70)** ^c^	**2.31 (1.29, 4.21)** ^c^	**< 0.01** ^c^
	Other	23.4%	Ref	Ref	
Breastfeeding	Yes	36.0%	1.09 (0.68, 1.78)	1.28 (0.73, 2.27)	0.40
	No	34.0%	Ref	Ref	
Exposure to smoking	Yes	37.3%	1.13 (0.74, 1.73)	1.43 (0.86, 2.40)	0.17
	No	34.5%	Ref	Ref	
Day care attendance^b^	Yes	51.2%	**3.34 (2.18, 5.15)** ^c^	**2.88 (1.62, 5.20)** ^c^	**< 0.01** ^c^
	No	23.9%	Ref	Ref	
Siblings	No	25.6%	Ref	Ref	
	1	41.4%	**2.06 (1.27, 3.35)** ^c^	**2.72 (1.55, 4.86)** ^c^	**< 0.01** ^c^
	> = 2	46.0%	**2.47 (1.44, 4.29)** ^c^	**2.78 (1.47, 5.34)** ^c^	**< 0.01** ^c^
Vaccination	Full	29.7%	0.85 (0.50, 1.43)	**0.48 (0.25, 0.92)** ^c^	**0.03** ^c^
	Incomplete	47.2%	1.80 (0.98, 3.30)	1.64 (0.79, 3.38)	0.18
	No	33.2%	Ref	Ref	
Origin of specimen	From a public center	34.0%	Ref	Ref	
	From a private center	37.7%	1.17 (0.77, 1.78)	1.05 (0.62, 1.77)	0.85

^a^Ref means reference group

^b^Includes children who were supervised during the morning hours by an adult who also cared for at least 3 other children

^c^Statistically significant associations are marked in bold

^d^Adjusted for gender, age, nationality, breastfeeding, exposure to smoking, day care attendance, siblings, vaccination and origin of specimen

^¶^ *p*-values for multiple logistic regression model

### Risk factors for drug resistant strains

The risk factors associated with colonization of children by a drug resistant strain to penicillin (full and intermediate resistance) are presented in Table 3. The only factor that was associated with an increased risk of colonization by drug resistant strains to penicillin was the use of antibiotics within the previous month before specimen collection [3-fold increased risk, OR: 2.83 (95% CI 1.09, 7.74)]. Two factors that appeared to be protective against colonization by resistant strains were female gender, [2.5 times lower risk, OR: 0.39 (95% CI 0.16, 0.90)], and the origin of the specimen from the office of a private paediatrician [3 times lower risk, OR: 0.32 (95% CI 0.12, 0.82)]. Percentages of resistance to penicillin are also presented in Table 3.

**Table 3:** Risk factors associated with colonization by a penicillin non-susceptible strain

Variable/Risk factor	Category	Percentage of resistance^a^ (%)	Unadjusted OR (95% CI)	Adjusted OR^d^ (95% CI)	*p*-value^¶^
Gender	Boy	48.7%	Ref ^b^	Ref ^b^	
	Girl	32.8%	0.51 (0.26, 1.01)	**0.39 (0.16, 0.90)** ^c^	**0.03** ^c^
Age	< 1 year	44.1%	Ref	Ref	
	1–2	47.4%	1.14 (0.45, 2.91)	0.89 (0.23, 2.76)	0.85
	> 2 years	37.1%	0.75 (0.33, 1.73)	0.47 (0.14, 1.55)	0.22
Nationality	Both parents Cypriots	41.1%	0.91 (0.40, 2.09)	0.93 (0.29, 3.03)	0.91
	Others	43.3%	Ref	Ref	
Breastfeeding	Yes	41.7%	1.10 (0.50, 2.48)	1.36 (0.52, 3.76)	0.54
	No	39.4%	Ref	Ref	
Exposure to smoking	Yes	43.4%	1.16 (0.58, 2.32)	1.45 (0.59, 3.57)	0.41
	No	39.8%	Ref	Ref	
Day care attendance	Yes	41.4%	1.03 (0.52, 2.06)	1.45 (0.54, 4.02)	0.47
	No	40.7%	Ref	Ref	
Siblings	No	41.9%	Ref	Ref	
	1	41.4%	0.98 (0.44, 2.19)	0.98 (0.35, 2.76)	0.97
	> = 2	40.0%	0.93 (0.38, 2.23)	0.67 (0.21, 2.06)	0.49
Vaccination	Full	29.6%	0.63 (0.23, 1.59)	0.81 (0.24, 2.63)	0.72
	Incomplete	56.0%	1.91 (0.77, 4.85)	2.55 (0.82, 8.32)	0.11
	No	40.0%	Ref	Ref	
Origin of specimen	From a public center	47.6%	Ref	Ref	
	From a private center	33.3%	0.55 (0.27,1.09)	**0.32 (0.12, 0.82)** ^c^	**0.02** ^c^
Antibiotics last month	Yes	60.6%	**2.86 (1.29, 6.55)** ^c^	**2.83 (1.09, 7.74)** ^c^	**0.04** ^c^
	No	35.0%	Ref	Ref	
Antibiotics last trimester	Yes	48.5%	1.80 (0.91, 3.63)	2.24 (0.95, 5.48)	0.07
	No	34.3%	Ref	Ref	

^a^Strain with intermediate and full resistance to penicillin are included

^b^Ref: means a reference group

^c^Statistically significant associations are marked in bold

^d^Adjusted for gender, age, nationality, breastfeeding, exposure to smoking, day care attendance, siblings, vaccination, origin of specimen and antibiotic use the last month

^¶^ *p*-values for multiple logistic regression model

In Table 4 we present identified risk factors associated with drug resistant isolates (full and intermediate resistance) to erythromycin. Univariable as well as multivariable analyses showed that the administration of antimicrobials to a child within the previous 30 days or the previous 3 months before specimen collection, both increased the risk of colonization by at least 3-fold (OR: 3.00 95% CI 1.15, 8.17). However, obtaining the specimen from a private paediatrician’s office versus the public sector outpatient clinic was associated with a protective effect for colonization [2.8 times lower risk, OR: 0.36 (95% CI 0.13,0.93)].

**Table 4:** Risk factors associated with carriage by an erythromycin non–susceptible strain

Variable/risk factor	Category	Percentage of resistance^a^ (%)	Unadjusted OR (95% CI)	Adjusted OR^d^ (95% CI)	*p*-value^¶^
Gender	Boy	43.6%	Ref ^b^	Ref ^b^	
	Girl	37.5%	0.77 (0.39, 1.52)	0.74 (0.32, 1.70)	0.48
Age	< 1 year	41.2%	Ref	Ref	
	1–2	44.7%	1.16 (0.45, 2.97)	1.39 (0.46, 4.31)	0.56
	> 2 years	35.7%	0.79 (0.34, 1.86)	0.92 (0.28, 3.02)	0.89
Nationality	Both parents Cypriots	41.1%	1.39 (0.61, 3.36)	3.04 (0.94, 10.70)	0.07
	Others	33.3%	Ref	Ref	
Breastfeeding	Yes	41.7%	1.43 (0.64, 3.33)	2.27 (0.84, 6.65)	0.12
	No	33.3%	Ref	Ref	
Exposure to smoking	Yes	41.5%	1.13 (0.56, 2.26)	1.62 (0.66, 4.00)	0.29
	No	38.6%	Ref	Ref	
Attendance at a Day care	Yes	39.1%	0.93 (0.47, 1.88)	1.04 (0.39, 2.80)	0.94
	No	40.7%	Ref	Ref	
Siblings	No	48.8%	Ref	Ref	
	1	39.7%	0.69 (0.31, 1.53)	0.65 (0.24, 1.76)	0.40
	≥2	30.0%	0.44 (0.18, 1.09)	**0.25 (0.07, 0.80)** ^c^	**0.02** ^c^
Vaccination	Full	29.6%	0.60 (0.22, 1.50)	0.34 (0.10, 1.08)	0.07
	Incomplete	44.0%	1.12 (0.44, 2.78)	0.66 (0.21, 2.05)	0.48
	No	41.3%	Ref	Ref	
Origin of specimen	From a public center	42.7%	Ref	Ref	
	From a private center	35.0%	0.73 (0.36,1.43)	**0.36 (0.13,0.93)** ^c^	**0.04** ^c^
Antibiotics last month	Yes	54.5%	**2.23 (1.01, 5.01)** ^c^	**3.00 (1.15, 8.17)** ^c^	**0.02** ^c^
	No	35.9%	Ref	Ref	
Antibiotics last trimester	Yes	51.5%	**2.66 (1.32, 5.47)** ^c^	**3.78 (1.56, 9.80)** ^c^	**< 0.01** ^c^
	No	30.0%	Ref	Ref	

^a^Strains with intermediate and full resistance to erythromycin are included

^b^Ref means a reference group

^c^Statistically significant associations are marked in bold

^d^Adjusted for gender, age, nationality, breastfeeding, exposure to smoking, day care attendance, siblings, vaccination, origin of specimen and antibiotic use the last month

^¶^*p*-values for multiple logistic regression model

## Discussion

To our knowledge, this is the first study in Cyprus to examine the association of various potential risk factors for carriage of *S. pneumoniae* and also for carriage of drug resistant isolates. Attendance at a day care center was identified as a risk factor for colonization by nearly 3-fold increased risk. Other studies have also reported similar findings and this is maybe due to the increased exposure of children to other children colonized or even suffering from a pneumococcal upper respiratory tract infection [9, 17]. Having siblings in the family has also been identified as a risk factor in the multi-variable analyses. Even one sibling increased the risk by at least 2.5 times. Other studies have also reported similar findings, which may be attributed to additional exposure to carriage or infections from siblings [9, 18].

Children with both parents originating from Cyprus had a significantly increased risk of colonization by *S. pneumoniae* isolates. The risk was increased by more than two times. This finding requires further investigation however, a possible explanation could be that these children may have a higher socioeconomic status compared to children from immigrants or other visitors due to the unique conditions of healthcare access in Cyprus discussed above. Higher socioeconomic status has been found among the risk factors associated with higher carriage of *S. pneumoniae* in one study from Greece [19].

Factors such as age, gender, history of breastfeeding, and passive exposure to smoking do not appear to be statistically significantly associated with the risk of colonization with *S. pneumoniae.* On the other hand, full immunization with PCV7 in comparison to non vaccinated children appeared to have a protective effect against colonization (two-fold decreased risk). Because of the herd effect, non immunized children can also benefit from the PCV7 vaccine. However the comparison between the groups revealed a protective effect of the vaccine against colonization. This has not been shown in other studies as in most countries the situation before and after the introduction of a conjugate vaccine is usually compared.

As shown in Tables 3 and 4, the most consistent and common factor associated with carriage of drug resistant isolates to either penicillin or erythromycin was the previous use of antibiotics during the month prior to specimen collection, raising the risk by more than 3 times. Use of antimicrobials even at 3 months prior to specimen collection was also a risk factor for colonization by drug resistant strains to both penicillin and erythromycin (2 to 3-fold increased risk). Many studies support our findings and have reported similar associations [10, 20, 21]. The association between high antibiotic use and high levels of antibiotic resistance in an area or a country is well documented [22, 23]. It is well known that the sub-inhibitory concentrations of anti-microbials allow *S. pneumoniae* to exhibit increased mutation frequency. Such increase in mutation frequency may give the bacterium a selective advantage as it helps to cope with the stress induced by the antimicrobials [24]. Selection of non-susceptible strains in colonization is likely to occur also by favoring the Growth and acquisition of non-susceptibility. Furthermore, horizontal gene transfer is considered a major cause of adaptability and spread of resistance genes within pneumococci and other mitis Group streptococci [25].

Female gender has also been found as an independent protective factor against carriage of penicillin drug resistant strains in comparison to males. Higher frequency of carriage with penicillin drug resistant pneumococcal isolates in boys was also detected in other studies however, no specific explanation has been identified [26]. Nevertheless, the frequency of pneumococcal infections in boys has also been reported to be higher than those in girls in various studies [27–29].

In our study, obtaining the specimen from a child who visited the private sector has been found to be an independent protective factor for carriage of either penicillin or erythromycin drug resistant isolates. This may reflect the dual health care system in Cyprus. It is possible that it could be explained by the difference in socioeconomic level between the two categories of children attending the private versus the public sector. The children visiting paediatricians in the private sector usually come from a higher socioeconomic status. In previous studies, higher socioeconomic level was also associated with lower carriage of pneumococci resistant to penicillin [18].

Our sample of children enrolled in this study, despite being a convenience sample, is largely representative of the general population of this age group. Children enrolled in the study come from the public and the private sector, therefore children from a broad socioeconomic spectrum participated in the study. Further to the above, children who attended a great number of day care centers and many others who were cared for at home are also represented. We believe that this broad representation prevented the cluster effect that would occur if we had included children from only a small number of day care centers.

High immunization coverage with PCV vaccines has been shown to decrease the frequency of invasive infections in general as well as by drug resistant isolates. This phenomenon is attributed to the significant decrease in the presence of pneumococcal serotypes included in the vaccine as many of these serotypes are characterized by significant resistance to antimicrobials [6]. As it has been shown in our published study, two of the most frequent serotypes colonizing Cypriot children, specifically 6B and 19F, are vaccine serotypes and demonstrated a high percentage of resistance to both penicillin and erythromycin [13]. In Cyprus, coverage with the PCV7 vaccine at the time of the study was relatively low [12]. In addition, antibiotic consumption in Cyprus is one of the highest in Europe [30] and antimicrobial resistance of *S. pneumoniae* to the main classes of antimicrobials in Cyprus has been found to be relatively high. In this study, multi-variable analyses revealed recent antibiotic use as the most consistent independent risk factor associated with increased risk of carriage for drug resistant pneumococcal strains to both penicillin and erythromycin. A campaign targeting the prudent use of antibiotics by physicians and the population in general, in combination with a vaccination campaign to increase the coverage of children by the available pneumococcal conjugate vaccines, may be the most important interventions towards the reduction of resistance of *S. pneumoniae* to anti-microbials and the lowering of the burden of these infections in Cyprus.

## Conclusions

Prudent use of antibiotics especially for upper respiratory tract infections in children as well as increased vaccination coverage by the pneumococcal conjugate vaccines could prove effective in reducing levels of colonization by non susceptible pneumococcal strains.

## Abbreviations

CI: Confidence Intervals)
CLSI: Clinical and Laboratory Standards Institute)
EU: European Union)
MIC: Minimum Inhibitory Concentration)
OR: Odds Ratio)
PCV7: Pneumococcus 7-valent conjugate vaccine)
